# Present but Overlooked: A Scoping Review of Instruments and Approaches for Measuring Presenteeism Related to Alcohol, Tobacco, and Drug Use

**DOI:** 10.1007/s10926-025-10317-z

**Published:** 2025-07-31

**Authors:** Kirrilly Thompson, Md Abdul Ahad, Gianluca Di Censo, Sonia Hines, Nicholas Rich, Alice McEntee, Jacqueline Bowden

**Affiliations:** 1 The National Centre for Education and Training on Addiction, Flinders University, Adelaide, South Australia Australia; 2 College of Medicine and Public Health, Flinders Health and Medical Research Institute, Flinders University, Adelaide, SA 5000 Australia; 3 School of Medicine and Public Health, College of Health, Medicine and Wellbeing, University of Newcastle, Callaghan, NSW 2308 Australia; 4 Department of Rural Sociology and Development, Sylhet Agricultural University, Sylhet, 3100 Bangladesh; 5 The University of Adelaide, JBI, School of Public Health, Adelaide, South Australia Australia

**Keywords:** Presenteeism, Alcohol and other drugs, Measurement, Instruments, Productivity, Workplace

## Abstract

**Purpose:**

The use of alcohol, tobacco, and other drugs (ATOD) can impact workplace productivity. Whilst presenteeism has a greater impact on productivity than absenteeism, it is less visible and often receives less attention. Measuring ATOD-related presenteeism is important for identifying the impact of AOD use and evaluating workplace AOD interventions. However, there is no standard approach to determining ATOD-related presenteeism. The aim of this scoping review was therefore to identify and describe different approaches and instruments used to determine ATOD-related presenteeism.

**Methods:**

A scoping review of publications up to and including December 2024 was undertaken across three major databases: Scopus, Ovid Medline, and the Latin-American and Caribbean System on Health Sciences (LILACS). The population was workers for whom ATOD-related presenteeism was reported, the concept was presenteeism, and the context was the workplace.

**Results:**

The review included 27 original studies. The most common approach (*n* = 22 studies) was *indirect*, which involved examining differences in presenteeism between workers who did and did not use ATOD. *Direct* approaches—asking participants explicitly about their ATOD-related presenteeism—were less common (*n* = 5 studies) and focussed exclusively on alcohol. Across both approaches, there was substantial variation in instruments (*n* = 4 direct, *n* = 10 presenteeism, *n* = 18 ATOD), use of validated instruments, recall periods, and ways of reporting findings, which may compromise the interpretation and synthesis of studies.

**Conclusion:**

This scoping review provides an evidence base for informing approach and instrument selection. It establishes the need for further research on the impact of different approaches and instruments on findings. This information is essential to encourage more rigorous and standardised approaches to determining ATOD-related presenteeism and evaluating workplace interventions.

## Background

The use of alcohol, tobacco, and other drugs (ATOD) is a significant global health concern. In 2019, alcohol consumption was associated with 2.6 million deaths [1]. The global burden of disease and injury caused by alcohol consumption can be quantified for 31 health conditions [1] and is a component cause of more than 200 health conditions and diseases [2]. The use of tobacco resulted in the deaths of over 8.7 million people in 2019 [3], and smoking has been causally linked to a wide range of diseases [4], whilst psychoactive drugs accounted for almost 600,000 deaths [1]. In addition to the negative health impacts of ATOD use at the population level, there are specific implications for worker health [5, 6] and productivity [6, 7]. For example, alcohol use can contribute to workplace accidents and injuries [8] through intoxication, hangover effects, and symptoms of withdrawal from problematic use. The impacts of tobacco use include workers taking breaks to smoke [9] and increased fire risk [10]. Illicit drugs and prescription or over-the-counter pharmaceuticals can impair workers physically, mentally and behaviourally, with direct effects on the safety of individuals, teams, and clients/the public [11].

When ATOD use hinders productivity and performance, there can be considerable expenses for employers and an adverse impact on the economy [7, 12, 13]. For example, one study [14] found that 21% of all adult workers across 32 American states were “binge” drinkers. Inefficiency due to drinking the previous day was reported by 11% of participants in a survey of Norwegian workers [15]. The cost of alcohol-related lost productivity amongst the working population of New Zealand has been estimated at $57 million per year [16]. In the Netherlands, 33% of employees reported impaired work performance as a result of ATOD use, and the associated cost was estimated at $2.63 billion [13]. The latest Australian data suggest that 36% of employed individuals exceed the national alcohol guidelines (i.e. drink at ‘high-risk’ levels), 18% are current tobacco smokers, and 19% used an illicit substance in the past year [17]. Workers in Australia who consume alcohol have been estimated to cost workplaces $3.9 billion annually in alcohol-related absenteeism alone [18]. The economic impact of lost productivity due to tobacco has been estimated at $388 billion over the working life of the population [19], and illicit drug-related absenteeism has been estimated to cost workplaces at least $2.9 billion annually [18].

There are two distinct ways in which ATOD use can impact workplace productivity. The first is when workers take leave from work, which is known as absenteeism. The second is presenteeism, which involves workers being physically present at work but not sufficiently well or fit to undertake their duties to their usual capacity. This can occur in situations such as being intoxicated, hungover or otherwise impaired by ATOD use [7]. Whilst absenteeism and presenteeism both result in lost productivity and increase the workloads of other workers [20], the frequency and cost of presenteeism usually exceed absenteeism [15, 21, 22]. One study, for example, estimated that the average annual cost of presenteeism attributable to alcohol use was over four times higher than costs related to absenteeism [23]. Presenteeism also has particular implications for worker health and safety, as it can exacerbate existing health issues, increase the risk of further illness, and compromise the safety of workers and their colleagues—especially in safety-sensitive industries [24, 25]. For these reasons, Kinman suggests that presenteeism should be seen as a risk-taking behaviour that requires careful measurement and management [24].

Whilst many studies have presented findings about the prevalence and cost of ATOD-related presenteeism (Table 1), a preliminary review of the literature suggested that there was a lack of consistency in the measurement of ATOD-related presenteeism, as is the case with presenteeism in general [26, 27]. Moreover, to our knowledge, there has been no review of ATOD-related presenteeism instruments, including the extent to which standard approaches may or may not be in use, as well as their validity and reliability. This information is important for addressing the accuracy of presenteeism determinations, which is necessary for making informed decisions about prioritising and evaluating ATOD interventions for worker safety, health and wellbeing. Therefore, the aim of this scoping review was to identify and describe instruments and approaches that have been used to measure ATOD-related presenteeism.

**Table 1:** Characteristics of included studies

Author, year	Primary aim/purpose	Country	Study design	Total number of participants	Type of worker/occupation and sector	Type of substance considered
1. Aas 2017 [28]	To explore the influence of annual drinking frequency and binge [sic] drinking on sickness presenteeism and impaired daily activities in a sample of Norwegian employees	Norway	Cross-sectional	3278	Production worker, transport worker, hotel/restaurant worker, administrative worker, and healthcare worker	Alcohol
2. Baker 2017 [29]	To expand the understanding of the work-related burden associated with smoking and quitting across the US, the European Union (EU), and China	United States of America, United Kingdom, France, Germany, Italy, Spain, China	Cross-sectional	126,904	Unspecified	Tobacco
3. Baker 2018 [30]	To quantify the difference in indirect costs associated with work productivity between current smokers and former smokers categorized by years since quitting smoking	United States of America	Cross-sectional	15,181	Unspecified	Tobacco
4. Bonsaksen 2021 [31]	To explore associations between alcohol-related presenteeism and (i) levels of psychological demands, decision latitude and support, (ii) levels of perceived effort, reward and overcommitment at work, (iii) perceived effort-reward imbalance, (iv) a high-strain job, low perceived support, high effort-reward imbalance and high over commitment, and (v) accumulated work environment risk factors	Norway	Cross-sectional	6620	Regular employee, middle management, top executive, and other	Alcohol
5. Bunn Iii 2006 [32]	To describe the frequency and cost of health-related productivity losses due to absenteeism and presenteeism based on self-reported smoking status in a large, cross-sectional database of employees throughout the United States	United States of America	Cross-sectional	34,934	Managerial and administrative	Tobacco
6. Buvik 2018 [15]	To map the prevalence of alcohol-related absence and inefficiency amongst employees in Norway, and to explore how absence and presenteeism due to alcohol use are experienced and handled at the workplace	Norway	Cross-sectional	1940	Employee from media, public administration, research and education,	Alcohol
7. França 2022 [33]	To assess individual and population-level estimates of work loss and economic costs due to mental and substance use disorders, and to assess the magnitude of their associations in the economically active population living in the *São* Paulo Metropolitan Area	Brazil	Cross-sectional	3007	Unspecified	Alcohol and illicit drugs
8. Goetzel 2009 [34]	To evaluate the impact of health risk factors on health and productivity expenditures within a single large U.S. employer, and to quantify differences in these outcomes, comparing high-risk employees to those at lower risk	United States of America	Cross-sectional	5875	Pharmaceutical company employees	Alcohol and Tobacco
9. Haglund 2015 [35]	To examine predictors of presenteeism and activity impairment and the possible associations between presenteeism and activity impairment outside work in a defined spondyloarthritis (SpA) cohort in southern Sweden. Secondary aims were to study possible differences in presenteeism and activity impairment with regard to gender and SpA subgroups	Sweden	Longitudinal	1253	Unspecified	Tobacco
10. Henke 2020 [36]	To measure the prevalence of opioid use disorder (OUD) and employee health care and productivity costs with and without OUD and to assess whether utilization of pharmacotherapy for OUD reduces those costs	United States of America	Cross-sectional	972,936	Unspecified	Opioids and non-opioid use (unspecified)
11. Holden 2011 [37]	To describe the impact on absenteeism and presenteeism for a range of health conditions in a large sample of working Australians	Australia	Cross-sectional	78,430	Industry employees (health, education, government, and finance)	Alcohol and illicit drugs
12. Lee 2021 [38]	To explore the impacts of smoking and alcohol consumption on workplace presenteeism based on demographic, health-related, and employment-related variables	South Korea	Cross-sectional	41,404	Blue collar (skilled and semi-skilled, elementary workers, agricultural, forestry, and fishery	Alcohol and Tobacco
13. Musich 2006 [39]	To investigate the impact of selected corporate environment factors, health risks, and medical conditions on job performance using a self-reported measure of presenteeism	Australia	Cross-sectional	1523	Senior manager, manager, line manager, and non-manager	Alcohol; Tobacco; Other: Drugs/medication used for relaxation
14. Otsubo 2024 [40]	To examine the association between presenteeism and subjective sleep quality, smoking status, and alcohol consumption	Japan	Cross-sectional	713	Office workers	Alcohol and Tobacco
15. Pelletier 2004 [41]	To examine the association between changes in health risks and changes in productivity over time	United States of America	Longitudinal	500	Unspecified	Alcohol and Tobacco
16. Santos 2008 [42]	To assess the association between habitual physical activity score and some outcomes associated with individual health, such as use of health services, absenteeism, and presenteeism	Brazil	Cross-sectional	376	Unspecified	Alcohol and Tobacco
17. Severeijns 2024 [13]	To evaluate the rate of absenteeism and presenteeism due to alcohol hangover in 2019	Netherlands	Cross-sectional	347	Unspecified	Alcohol
18. Sherman 2013 [43]	To quantify and compare more completely the health- and productivity-associated costs for smokers and nonsmokers amongst the workforce of a single employer	United States of America	Cross-sectional	14,387	Unspecified	Tobacco
19. Shiratsuchi 2024 [44]	To examine the relationship between occupational stress and presenteeism status amongst employees in both small and medium enterprises	Japan	Cross-sectional	554	Unspecified	Alcohol and Tobacco
20. Shrestha 2022 [45]	To estimate the cost of morbidity-related productivity losses in 2018 (absenteeism, presenteeism, inability to work, and household productivity losses) attributable to cigarette smoking	United States of America	Cross-sectional	83,046	Management, production, or service related	Alcohol and Tobacco
21. Sullivan 2019 [23]	To estimate the cost of lost productivity associated with alcohol use in New Zealand and to describe and quantify its impact on employers	New Zealand	Cross-sectional	1027 (800 employees and 227 employers)	Senior manager/director, Junior/middle management, Foreperson/supervisor	Alcohol
22. Suwa 2017 [46]	To show the benefits of smoking cessation for workplace productivity and decreased costs associated with loss of work impairment	Japan	Cross-sectional	23,738	Unspecified	Tobacco
23. Thompson 2021 [47]	To investigate the association between worklife prevalence measures of presenteeism and lifetime prevalence of twelve psychosocial vulnerabilities, encompassing mental health, mental health-related, and addictive conditions	Canada	Cross-sectional	2817	Manager/Professional Services	Alcohol, Tobacco and Illicit drugs
24. Tran 2018 [48]	To explore the health-related work productivity loss between rural and urban methadone maintenance programme, patients in Vietnam and identify associated factors	Vietnam	Cross-sectional	1016	Unspecified	Alcohol, Tobacco, and Opioids
25. Tsuchida 2024 [49]	To examine the relationship between changes in lifestyle habits and presenteeism change according to sex	Japan	Longitudinal	9366	Managers and non-managers	Alcohol and Tobacco
26. Williden 2012 [50]	To investigate the associations between health risk factors, absenteeism, and presenteeism in the New Zealand population	New Zealand	Cross-sectional	747	Unspecified	Alcohol and Tobacco
27. Won 2022 [51]	To evaluate how past presenteeism affects future absence by evaluating absence 1 year after evaluating presenteeism	Korea	Longitudinal	12,572	Non-white collar workers	Alcohol and Tobacco

## Methods

A scoping review was conducted, following JBI scoping review methodology [52] and in accordance with a protocol registered with the Open Science Framework (OSF) (Registration: 10.17605/OSF.IO/S83Y5). Findings are reported according to the 2020 Preferred Reporting Items for Systematic Reviews and Meta-Analyses (PRISMA) guideline.

### Eligibility criteria

The selection of studies was informed by the population, concept, and context (PCC) framework [53]. The population was workers for whom ATOD-related presenteeism was reported, the concept was presenteeism, and the context was the workplace. This review included studies published in English, Spanish, and Portuguese, of which the latter were translated using Google Translate and supplemented by the Romance language skills of GD and KT (Italian and Spanish). Studies were included if they included workers 18 years and over who had used at least one type of ATOD, provided information about how presenteeism was measured, presented data on presenteeism due to the use of at least one type of substance, and were based on self-report measures. Eligible study types were peer-reviewed publications, dissertations, and conference proceedings. No limitations were imposed on earliest year of publication or type of workplace/occupation.

### Search strategy

Three databases were searched, including Scopus, Ovid Medline, and the Latin and Caribbean Literature on Health Sciences (LILACS) which includes documents in English, Spanish, and Portuguese. In addition, grey literature was searched through Google Advanced and ProQuest Dissertations and Theses. A general desktop search was also undertaken in Google Scholar (limited to the first 10 pages). Database searches were completed in July 2023 without any language or time restrictions and subsequently updated in August 2024. The reference lists of all included studies were searched for other eligible studies for inclusion. The search strategy was informed by initial unrecorded background searches in Scopus and Google Scholar (limited to the first 50 documents) to identify relevant search terms, keywords, and controlled vocabulary. We identified key words related to workplace, presenteeism, ATOD, and combined them with appropriate Boolean operators to generate index and search terms. Assistance was provided by an experienced university librarian, particularly in relation to approving search terms.

### Study selection

Studies were imported into Endnote and de-duplicated before being imported into Covidence. Initial title and abstract screening were completed by three reviewers (NR, KT, and SH), and disagreements were resolved through discussion. Studies added after the updated search were screened by two reviewers (MAA and GD) with consensus undertaken by a third reviewer (KT).

### Data extraction, collation, and interpretation

A standardized data extraction chart was designed to address the study aims, based on existing relevant studies and discussions amongst the authors of this scoping review. Data were extracted on the characteristics of the study (author/s, year of publication, title, country, study aim, type of evidence, type of participant [worker, supervisor]) and sample size, organisation/sector, type of substance, type of instrument/measure [established or self-designed], name of the instruments, questions used to measure presenteeism, questions or instruments related to at least one type of ATOD use, and period of recall. The data extraction chart was piloted with four studies and refined accordingly. Extraction was completed by two reviewers (MAA and GD) with consensus undertaken by a third reviewer (KT). The extraction chart was exported from Covidence into an excel spreadsheet for the purposes of summarising findings with descriptive statistics and presenting them using a narrative synthesis approach [54]. Literature was searched separately for evidence of instrument validation. Income classification of countries was conducted against the World Bank country classifications [55].

## Results

**Fig. 1:**
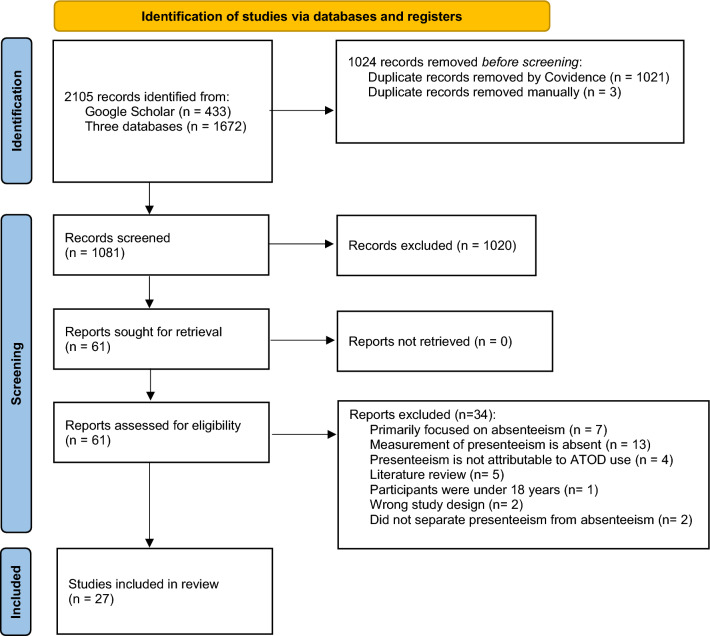
PRISMA flow diagram of the included studies

Searches yielded 2105 records, of which 1672 were retrieved from Scopus, Ovid Medline, ProQuest (Dissertation), and LILACS, whilst 433 were identified from Google Scholar. A total of 1024 duplicate records were removed. The remaining 1081 records underwent title and abstract screening, and 61 were eligible for full-text review. After full-text review, 27 studies (Table 1) [13, 15, 23, 28–51] met eligibility for inclusion. Studies were excluded if they focussed primarily on absenteeism (and did not include presenteeism), presenteeism was not reported in relation to ATOD use, participants were under 18 years of age, or if presenteeism was aggregated with absenteeism (See the PRISMA flow diagram, Fig. 1).

### Characteristics of eligible studies

The characteristics of the 27 included studies are detailed in Table 1.

#### Study type, design, and sample size

Almost all (*n* = 26, 96%) studies were peer-reviewed research articles published in English [13, 15, 23, 28–41, 43–51] with one (*n* = 1, 4%) a thesis [42] published in Portuguese. Studies were published over a 20-year period spanning 2004 to 2024, with five (19%) [32, 34, 39, 41, 42] published before 2010. A cross-sectional study design was employed in most studies (*n* = 23, 85%) [13, 15, 23, 28–40, 42–48, 50, 56] with four (15%) [35, 41, 49, 51] utilising a longitudinal design. Almost all studies employed quantitative methods and one study (4%) employed a mixed-methods design combining surveys with interviews [15]. Over one quarter of studies (*n* = 7, 26%) conducted secondary data analyses, primarily using data from national survey data sources [29, 30, 36, 38, 45, 46, 49]. Sample sizes ranged from 347 to 972,936 participants. Three studies (11%) [13, 42, 56] involved 500 or fewer participants, whilst nine (33%) [29, 32, 36–38, 43, 45, 46, 51] involved more than 10,000 participants (Table 1).

#### Country

Most studies (*n* = 24, 89%) were conducted in high-income countries. Two studies (7%) were conducted in the upper middle income country of Brazil [33, 42], and one (4%) was conducted in the lower middle income country (LMIC) of Vietnam [48] (Table 1).

#### Type of workers and industries

Almost half the studies (*n* = 13, 48%) did not specify the type (role or occupation) of workers examined. Of the 14 that did, 12 (86%) reported data from general employees/workers [28, 31, 32, 34, 37–40, 45, 47, 49, 51] and seven (50%) from managers/employers [23, 31, 32, 39, 45, 47, 49].

Twenty-two (81%) studies did not report type of sector/industry. Of the five (19%) that did, workers were from education [15, 37, 38], media [15], finance [37], public administration [15], hospitality [28], and research [15] as well as the following industries that can be considered safety-sensitive: agriculture [38], forestry and fishing [38], pharmaceuticals [34], production [28], transport [28], and health care [28] (Table 1).

#### Study aims and substances considered

Over half of the studies (n = 15, 56%) included presenteeism and at least one substance type amongst their primary aim [13, 15, 23, 28–33, 36, 38, 40, 43, 45, 46]. The other 12 (44%) studies were primarily concerned with presenteeism in relation to general health [34, 37, 39, 41, 48, 50], or specific health-related topics such as spondyloarthritis [35], physical activity [42], occupational stress [44], psychosocial vulnerability [47], lifestyle habits [49], and previous performance [51].

Almost half of the studies (*n* = 13, 48%) [34, 38–42, 44, 45, 47–51] examined presenteeism related to at least alcohol and tobacco. Six studies (22%) [29, 30, 32, 35, 43, 46] examined tobacco-related presenteeism exclusively, five (19%) examined alcohol-related presenteeism exclusively [13, 15, 23, 28, 31]. No studies examined illicit drugs exclusively, but three studies (11%)—which were undertaken in Australia [37], Brazil [33], and Canada [47]—included the use of illicit substances. Two studies (7%) in the United States of America [36] and Vietnam [48] considered the use of opioids. One study (4%) included drugs/medication used for relaxation [39] (Table 1).

### Direct approaches

Five (19%) studies reported ATOD-related presenteeism based on a direct approach whereby participants were asked to report on presenteeism explicitly [13, 15, 23, 28, 31] (Table 2). These studies considered presenteeism related exclusively to alcohol consumption. Three studies using the direct approach were undertaken in Norway [15, 28, 31], with the others in the Netherlands [13] and New Zealand [23].

**Table 2:** Instrument used to measure ATOD associated presenteeism directly

Instrument	Validation Y/N	Included studies using this instrument	Relevant question/s	Number and type of relevant questions	Recall period	Reported as
*Work-related instruments*						
1. The Work Productivity and Activity Impairment Questionnaire (General Health) (WPAI-GH) [57]	Y [57]	*n* = 2 [28, 31]	1. During the past seven days, how much did alcohol consumption affect your productivity whilst you were working? [28] 2. Whilst working during the last seven days, how much did your alcohol consumption impact on your productivity? [31]	One question as an 11-point visual analogue scale [28] or an 11-point Likert scale [31] (i.e. 0–10).	Past week [28, 31]	1. Extent of impact was measured based on a point scale measures (0 to 10), with 0 indicating no loss of productivity and 10 indicating the highest loss of productivity
2. Health and Labour Questionnaire (HLQ) [58]	N	*n* = 1 [13]	Questions in the study concerned the number of days in 2019 of presenteeism, i.e. the number of days worked whilst having a hangover, and in comparison to a regular working day without having a hangover, how well they performed at work on days when they had an alcohol hangover	Two questions: open-ended numeric responses [13]	Previous year	1. Number of times 2. Extent of impact (from not affected to much worse performance than usual on a scale ranging from 0 to 100%)
*Self-designed instruments*						
3. Self-designed	N	*n* = 1 [23]	How many productive hours lost each week due to alcohol consumption?	One question: open-ended numeric response [23]	Past seven days	Number of unproductive hours
4. Self-designed	N	*n* = 1 [15]	Have you experienced inefficiency due to drinking alcohol?	One question: dichotomous responses (Yes/No) [15]	Past 12 months	Frequency (number and percentage) of workers reporting reduced productivity

#### Instruments directly measuring ATOD-related presenteeism

Four instruments were identified that directly measured ATOD-related presenteeism (Table 2). These included two established instruments and two self-designed questions. One established instrument has been validated – the Work Productivity and Activity Impairment Questionnaire (General Health) (WPAI-GH) [57]. Two [28, 31] studies adapted questions from the WPAI-GH specifically for alcohol (i.e. there are no questions directly linked with ATOD use in the WPAI-GH). One study [13] adapted existing alcohol-related questions from the non-validated Health and Labour Questionnaire (HLQ) [58], following an approach used by the Institute of Alcohol Studies [59]. Studies adopting the direct approach to measuring ATOD-related presenteeism included safety [28] and non-safety-sensitive industries [15]. Recall periods were either one week or one year, and findings were reported in terms of number of times, number of hours, and extent of impact.

### Indirect approaches

Most (*n* = 22, 81%) studies adopted an indirect approach to determining ATOD-related presenteeism by examining differences in presenteeism between individuals who did and did not use ATOD. This mostly necessitated the use of separate surveys to assess presenteeism and ATOD use, although three (14%) of the 22 studies using the indirect approach utilised separate questions on presenteeism and ATOD use from the same survey; the Korean Working Conditions Survey [38], the AHM Health Risk Appraisal [39] (AHM/HRA), and the Wellness Inventory [32]. Indirect approaches were used for all substances. There were no clear associations between instrument selection and type of substance or country, although the World Health Organization Health and Work Performance Questionnaire (WHO-HPQ) was used to determine presenteeism in two of the three studies considering the use of illicit drugs [37, 47].

#### Instruments used to determine presenteeism

Ten different presenteeism instruments were identified amongst the studies using an indirect approach to determine ATOD-related presenteeism (Table 3). This included five established work-related instruments, two established health-related instruments, and three self-designed instruments. One study did not detail how presenteeism was determined [45]. All established instruments (*n* = 7) were validated. The most commonly used instruments were the WHO-HPQ [60, 61] (*n* = 7 studies) composed of four questions [37, 40, 42, 43, 47, 49, 50], and the Work Productivity and Activity Impairment Questionnaire (General Health) (WPAI-GH) [57] (n = 6 studies) composed of one question [29, 30, 35, 41, 46, 48]. The WHO-HPQ was applied to studies of workers in both safety and non-safety-sensitive industries, but type of worker/occupation was unspecified for studies using the WPAI-GH for an indirect approach to determining ATOD-related presenteeism. Both of these instruments were used across diverse jurisdictions with no apparent patterns.

**Table 3:** Instruments used to determine presenteeism

Instrument	Validated Y/N	Included studies using this instrument	Relevant questions implemented by studies	Number and type of relevant questions	Recall period	Reported as
*Work-specific instruments*						
1. World Health Organization Health and Work Performance Questionnaire (WHO-HPQ) [60, 61]	Y [62]	*n* = 7 [37, 40, 42, 43, 47, 49, 50]	1. Using the 0 to 10 scale, how would you rate your usual work performance over the past year? [42] 2. Using the 0 to 10 scale, how would you rate your overall job performance on the days you worked during the past 4 weeks [28 days]? [37, 40, 42, 49, 50] 3. Using the same 0 to 10 scale, how would you rate your overall job performance on the days you worked during the past week [7 days]? [47] 4. On a scale from 0 to 10 where 0 is the worst job performance anyone could have at your job and 10 is the performance of a top worker, how would you rate the usual performance of most workers in a job similar to yours? [47]	Four questions: an 11-point Likert scale (0 = worst possible performance, 10 = best possible performance) [37, 40, 42, 43, 47, 49, 50]	Past 7 days [47], past month [37, 40, 42, 43, 49, 50], and past year [42]	Mean of decrease in work performance [37, 40, 42, 43, 47, 49, 50]
2. The Work Productivity and Activity Impairment Questionnaire (General Health) (WPAI-GH) [57]	Y – for various diseases [57, 63]	*n* = 6 [29, 30, 35, 41, 46, 48]	Adapted questions: 1. During the past seven days, not including today, how much did your health problems affect your productivity whilst you were working? [29, 30, 35, 41, 46, 48]	One question: numerical [29, 30, 35, 41, 46], and a 10-point scale [41, 48]	Past seven days [29, 30, 35, 41, 46, 48]	Percentage [29, 30, 35, 41, 46] or mean [48] of impact of reduced productivity at work
3. The Work Limitations Questionnaire [64]	Y [65]	*n* = 1 [34]	What is the number of unproductive days in past 14 days? [34]	One question: numerical response [34]	Past 14 days [34]	Number of unproductive days [34]
4. Second and Third Korean Working Conditions Survey [66]	Y (second version) [67]	*n* = 1 [38]	Have you ever come to work whilst sick in the past 12 months? [38]	One question: dichotomous responses (yes/no) [38]	Past 12 months [38]	Percentage of workers engaging in presenteeism [38]
5. The WHO Disability Assessment Schedule Version 2	Y [68]	*n* = 1 [33]	1. The number of days, in the previous 30, in which respondents were able to go to their workplace and carry out their other normal activities, but had to cut down on the amount of work they did, or did not get as much done as usual, because of health problems 2 The number of days, in the previous 30 days, in which respondents were able to go to their workplace and carry out their other normal activities but had to cut back on the quality of their work or on how carefully they worked, because of health problems [33]	Two questions: numerical response [33]	Past 30 days [33]	Number of unproductive days [33]
*Health-related instruments with presenteeism-related questions*						
6. The Wellness Inventory (WI) [69]	Y [70]	*n* = 1 [32]	What is the number of unproductive hours in past 12 months? [32]	One question: numerical [32]	Past 12 months [32]	Number of unproductive hours [32]
7. AHM Health Risk Appraisal (HRA) [71]	Y [72]	*n* = 1 [39]	How much time did your stress levels, physical or emotional health make it difficult for you to do the following: [1] Work your required number of hours; [2] Use your equipment properly (e.g. keyboard, mouse, tools, or machinery); [3] Concentrate on your work; [4] Work effectively with others; and [5] Work to the best of your ability? [39]	Five questions: numerical, and dichotomous (yes/no) (nominal) responses [39]	Past 4 weeks [39]	Number of unproductive hours [39]
*Self-designed instruments*						
8. Self-designed	N	*n* = 1 [51]	During the past year, have you ever worked despite being sick? [51]	One question: dichotomous response (yes/no) [51]	Past 12 months [51]	Percentage of workers engaging in presenteeism [51]
9. Self-designed	N	*n* = 1 [36]	In the past 4 weeks, how many days did your health problems affect productivity at work? [36]	One question: numerical response [36]	Past 4 weeks [36]	Number of unproductive days [36]
10. Self-designed	N	*n* = 1 [44]	Which health problems had the most significant impact on your work? [44]	One question: multiple choice [44]	Past four weeks [44]	Prevalence of health risk factors in presenteeism status [44]

Across presenteeism instruments, recall periods ranged from last seven days to past 12 months. Eight studies used recall periods of two weeks or less [29, 30, 34, 35, 41, 46–48], and recall periods used with the most common instrument—the WHO-HPQ—varied across studies. Units of measurement and reporting also varied, including number of unproductive hours or days, a scale of difficulty getting work done, hours worked whilst impaired, effect on productivity (numerical scale), self-ratings of job performance, and dichotomous responses to working whilst sick or affected by a health problem (yes or no) (Table 3).

#### Instruments used to determine ATOD use

Across the 22 studies using an indirect approach to determine ATOD-related presenteeism, 18 different instruments were identified to determine ATOD use (Table 4). This included four established ATOD-specific instruments, 10 established health-related instruments with ATOD questions, and four self-designed instruments. Eight of the established instruments were validated (57%). One study [42] used questions from two instruments, and five studies [29, 35–37, 51] did not report the instruments used to determine ATOD use. The most commonly used established instruments were the validated Alcohol Use Disorders Identification Test (AUDIT) [73], comprising 10 questions, and its shorter version the AUDIT-C [74], comprising three questions and used in two studies in Canada and Vietnam [47, 48], as well as the validated Health Risk Appraisal [71] comprising five questions and used in two studies in the USA and Australia [39, 41]. It is unclear if these studies included workers in safety or non-safety-sensitive industries.

**Table 4:** Instruments used to determine ATOD use

Instrument	Validated Y/N	Included studies using this instrument	Relevant question/s	Number and type of relevant questions	Recall period	Reported as
*ATOD-specific instruments*						
1. Alcohol Use Disorders Identification Test (AUDIT) [73] *and* Alcohol Use Disorders Identification Test-Consumption (AUDIT-C) [74]	Y [75], AUDIT classified as “excellent” [76]	*n* = 2 (AUDIT [47] and AUDIT-C [48]	AUDIT Too lengthy for inclusion in full. Please view questions at https://auditscreen.org/ AUDIT-C a) How often did you have a drink containing alcohol in the past year? b) On days in the past year when you drank alcohol how many drinks did you typically drink? c) How often did you have 6 or more (for men) or 4 or more (for women and everyone 65 and older) drinks on an occasion in the past year?	AUDIT Ten questions: categorical (never, less than monthly, monthly, weekly, and daily or almost daily) [47] AUDIT-C Three questions: numerical [48]	Currently, Past 30 days and Past 12 months [47, 48]	AUDIT: Frequency (number and percentage) [47] AUDIT-C: The minimum score is 0 (for non-drinkers), and the maximum possible score is 12 [48]
2. The Cut down drinking, Annoyed by criticism, Guilty feelings and Eye-opener (CAGE) questionnaire [77]	Y, classified as “excellent” [76]	*n* = 1 [34]	1. Have you ever felt you should cut down on your drinking? 2. Have people annoyed you by criticizing your drinking? 3. Have you ever felt bad or guilty about your drinking? 4. Have you ever had a drink first thing in the morning to steady your nerves or to get rid of a hangover? [34]	Four questions: dichotomous responses (yes, no) [34]	Lifetime [34]	Percentage of alcohol drinking and smoking [34]
3. Drug Abuse Screening Test (DAST) [78]	Y [79] DAST-10 and DAAST-20 classified as “excellent” [76]	*n* = 1 [47]	1. Do you abuse more than one drug at a time? [47]	One question: dichotomous responses (yes/no) [47]	Lifetime [47]	Frequency (number and percentage) [47]
4. Alberta Alcohol & Drug Abuse Commission (AADAC) (Adapted from National Alcohol Drug Abuse Survey, Canada) [80]	N	*n* = 1 [47]	1. Smoking within the past month, irrespective of the number of cigarettes consumed? [47]	One question: dichotomous responses (yes/no) [47]	Past 30 days [47]	Frequency (number and percentage) [47]
*Health-related instruments with ATOD-related questions*						
5. Health Risk Appraisal (HRA) [71]	Y [72]	*n* = 2—[41] and modified as the AHM HRA [39]	1. In the last 30 days, have you used tobacco? 2. Used a smokeless tobacco product? 3. In the past 7 days, on how many days did you drink alcohol? 4. On days when you drank alcohol, how often did you have __ (5 or more for men, 4 or more for women and those men and women 65 years old or over) alcoholic drinks on one occasion? 5. Do you ever drive after drinking, or ride with a driver who has been drinking? [Refer to Instrument^a^] [39, 41]	Five questions: dichotomous responses (yes/no), numerical, and categorical [39, 41]	Past 30 days (tobacco) past week and times per day (alcohol) [39, 41]	Percentage of alcohol drinking and smoking [39, 41]
6. Wellness Inventory [69]	Y [70, 81]	*n* = 1 [32]	1. Which cigarette smoking pattern best describes your behaviour? (never smoked, former smoker or current smoker) [32]	One question: categorical responses [32]	Past 12 months [32]	Frequency (number and percentage) [32]
7. INCA Behavioural Risk Factor Surveillance System (BRFSS) [82]	Y [83]	*n* = 1 [42]	1. How many times have you consumed alcoholic beverages in the past last THIRTY days? 2. Are you thinking about cutting down or stopping drinking? 3. Do you get upset if people criticize your drinking? 4. Do you feel bad or guilty about drinking alcohol? 5. Do you drink in the morning to calm down or ease a hangover? [42]	Five questions: numerical (open-ended) and dichotomous responses (yes, no) [42]	Past 30 days [42]	Frequency (number and percentage) [42]
8. World Mental Health Composite International Diagnostic Interview (WMH-CIDI 3.0) [61]	Y [84]	*n* = 1 [33]	Too lengthy for inclusion in full.^a^ The section on substance use screens for Alcohol Abuse, Alcohol Dependence, Drug Abuse, Drug Dependence, Nicotine Dependence [33]	Over 100 questions with skip logic: Numerical, scale, categorical	Past 12 months [33]	Percentage [33]
9. The Third National Health and Nutrition Examination Survey (NHANES III) [85]	N	*n* = 1 [42]	1. Have you smoked cigarettes in the last TWELVE months? 2. How many times have you smoked in the last THIRTY days? 3. For whom currently smokes: I smoke an average of __ cigarettes a day for __ years or __ months ago 4. For whom stopped smoking: I smoked an average of __ cigarettes a day for __ years, I quit __ years ago or __ months [42]	Four questions: dichotomous responses (yes, no) and numerical [42]	Past 12 months and 30 days [42]	Frequency (number and percentages) [42]
10. New Zealand Health Survey [86]	N	*n* = 1 [50]	1. Have you ever smoked a total of more than 100 cigarettes in your whole life? 2. How often do you now smoke? 3. How many drinks containing alcohol do you have on a typical day when you are drinking? [50]	Three questions: dichotomous responses (yes, no), categorical responses and numerical [50]	Tobacco (Lifetime for smoking more than 100 cigarettes, unspecified for other question) Alcohol (Past 12 months) [50]	Frequency (number and percentage) [50]
11. Second and Third Korean Working Conditions Survey (Adapted from Korean National Survey)[66]	Y (Second version) [67]	*n* = 1 [38]	1. Have you ever smoked in your lifetime? 2. Are you currently smoking? 3. How often do you drink alcohol? 4. How many glasses do you usually consume when you drink? [38]	Four questions: Dichotomous responses (yes/no), categorical, [38]	Lifetime [38]	Frequency (number and percentage) [38]
12. The 2014 − 2018 National Health Interview Survey (NHIS), United States [87]	N	*n* = 1 [45]	1. Have you smoked at least 100 cigarettes in your entire life? 2. Do you now smoke cigarettes every day, some days or not at all? 3. How long has it been since you quit smoking cigarettes? [45]	Three questions: Dichotomous responses (yes, no), and numerical (open-ended) [45]	Lifetime, Some days, Every day [45]	Frequency (number and percentage) [45]
13. Specific Health Check-up and Guidance programme [88]	N	*n* = 1 [49]	1. Do you smoke cigarettes regularly? 2. How often do you drink? [49]	Two questions: dichotomous response (yes, no) and three point scale [49]	Per day [49]	Frequency (number and percentages) [49]
14. The 2013 US National Health and Wellness Survey [89]	N	*n* = 1 [30]	1. Have you ever smoked cigarettes? 2. What is your current smoking status? 3. If you are a former smoker in what year did you quit smoking? [30]	Three questions, dichotomous responses (yes, no), categorical, numerical (open-ended) [30]	Current and lifetime [30]	Frequency (number and percentage) [30]
*Self-designed instruments*						
15. Self-designed	N	*n *= 1 [40]	1. What is your current smoking status? 2. What is your drinking status? [40]	Two questions: categorical responses [40]	Current and lifetime [40]	Frequency (number and percentage) [40]
16. Self-designed	N	*n* = 1 [43]	1. How would you describe your cigarette smoking habits? [43]	One question: categorical responses [43]	Lifetime [43]	Frequency (number and percentage) [43]
17. Self-designed	N	*n* = 1 [44]	1. Do you currently smoke cigarettes? 2. What is your alcohol consumption habit? [44]	Two questions: dichotomous responses (yes, no), categorical [44]	Current (smoking) and lifetime (alcohol) [44]	Frequency (number and percentage) [44]
18. Self-designed	N	*n* = 1 [46]	1. Have you ever smoked? 2. What is your current smoking status? [46]	Two questions: dichotomous responses (yes, no), categorical responses [46]	Current and lifetime use [46]	Frequency (number and percentage) [46]

There was a wide range of recall periods for most instruments, ranging from daily use to lifetime use. Lifetime use was particularly common for reporting tobacco use. The Alcohol Use Disorders Identification Test (AUDIT) was implemented with recall periods of 30 days [47, 48] and past 12 months [47]. The Health Risk Appraisal used a recall period of the past 30 days for tobacco and a recall period of the past week for the number of drinks consumed per occasion for alcohol [39]. Ways of reporting ATOD use also varied across studies, mostly to reflect dichotomous definitions of current or past smoker (yes/no), and continuous measures of frequency and quantity of alcohol consumption and/or risk scores of alcohol and other drug use (Table 4).

## Discussion

The impact of presenteeism on workplace productivity frequently eclipses that of absenteeism and ATOD use is a well-recognised contributor to presenteeism [15, 21, 22]. However, the approaches and instruments used to determine the impact of ATOD use on presenteeism have been largely overlooked, resulting in uncertainty of the accuracy of different figures. Moreover, given that ATOD use is modifiable through workplace interventions, evaluations rely on reliable measurement of ATOD-related presenteeism. The aim of this scoping review was therefore to identify and describe different approaches and instruments.

The most common approach to determining ATOD-related presenteeism across the studies was the indirect approach, which involved examining differences in presenteeism between workers who did and did not use ATOD. This may be considered unexpected given the fact that there are two obvious advantages to the direct approaches. First, direct approaches may result in more efficient surveys for workers and researchers alike by reducing the number of questions and steps required to complete surveys and analyse data. Second, the lack of ambiguity in direct questions may result in more valid data. For instance, the Institute of Alcohol Studies in the UK justified their decision to use a direct question in their national survey used by one of the studies in this review [13], ‘in the expectation that they are the best judges of their own performance’ whilst also acknowledging the limitations of self-report data [59].

However, the relative merits of using a direct approach to determining ATOD-related presenteeism may be outweighed by the broader context of socio-cultural, legal, and workplace policy considerations. For example, substance use is often stigmatised [90], illicit drug use is also by definition criminalised, and some workplace policies stipulate performance management, disciplinary action, or dismissal for workers found to be under the influence of substances [91]. These considerations can exacerbate workers’ concerns about confidentiality [92, 93] and related reluctance to disclose the use of illicit substances. Response validity of direct approaches is therefore likely to be biassed not only by social desirability but the financial imperative to maintain employment—especially in safety-sensitive industries where alcohol and other drug testing is routine or mandated [94] and zero tolerance policies may apply.

Whilst worker responses can be collected anonymously, by a third party, and/or with guarantees of confidentiality, the threat of stigma, performance management or dismissal can still understandably result in mistrust and suspicion of the intentions of employers or researchers. This can undermine even the most health-centred and non-punitive attempts to collect data from workers on ATOD use, such as where the aim is to inform the design and evaluation of workplace ATOD interventions [92, 93]. Together, these considerations may explain why indirect approaches were more common, and why alcohol and tobacco were the most considered substances across the studies included in this review. They may also explain the notable absence of the World Health Organization (WHO) Alcohol, Smoking and Substance Involvement Screening Test (ASSIST)[95] and the shorter ASSIST-lite [96], both of which can screen for all licit and illicit substances (tobacco, alcohol, cannabis, cocaine, amphetamine-type stimulants including ecstasy, inhalants, sedatives, hallucinogens, opioids, and ‘other drugs’).

Whilst not a perfect solution to mitigating the effects of self-report bias or stigma, indirect approaches do not require workers to comment directly on how their ATOD use has impacted their productivity. They also enable more complex data analysis by making it possible to examine whether presenteeism differs for other variables (e.g. sex, full-time vs part-time, type of job role, etc.) Moreover, indirect approaches offer advantages for the evaluation of workplace ATOD interventions whereby pre-post and longitudinal evaluations could identify significant differences on presenteeism but not ATOD use and *vice versa*. However, the methodological rigour of indirect approaches may be undermined by several factors identified in this review, such as issues related to instrument validity. Indeed, evidence of validation was only found for 13 (45%) of all 29 unique instruments identified in this review (established and self-made), and 13 (65%) of all 20 established instruments.

Encouragingly, the most commonly used instrument for the direct approach (the WPAI-GH) was validated and has been successfully adapted to consider specific ATOD-related presenteeism issues such as the impact of methadone maintenance treatment [48] and smoking dependence and alcohol use by onshore oil field workers [97]. For the indirect approach, all the established instruments used to determine presenteeism were validated—including the most used instruments (WHO-HPQ and WPAI). Moreover, the two most commonly used instruments used to determine ATOD use (AUDIT/AUDIT-C and the Health Risk Appraisal) were classified as psychometrically “excellent” in a 2023 systematic review of psychometric properties of brief, free, and readily accessible self-report measures of substance use and related factors [76]. However, three presenteeism instruments identified in a 2015 systematic review as having the strongest level of evidence on more than one measurement property [25] were not implemented in any of the studies included in the present scoping review (Stanford Presenteeism Scale—6-item version, the Endicott Work Productivity Scale, and the Health and Work Questionnaire). Although most presenteeism instruments in that earlier systematic review had been evaluated for construct validity, there was very little evidence for criterion validity [25]. Also, some of the studies in the present review adapted or selected questions from validated instruments (e.g. the use of AUDIT for alcohol but self-designed questions for tobacco [40]), when the selection of questions from a validated tool may not have the same properties as the whole tool. Finally, the combination of different presenteeism and ATOD instruments (or parts thereof) has not, to our knowledge, been validated as an approach to determining ATOD-related presenteeism.

Findings of the present scoping review also raise concerns regarding the diversity of recall periods for presenteeism and ATOD use identified in this review, such as where a study considers smoking over the previous 30 days but presenteeism over the past seven days [47]. Inconsistent recall periods may complicate the interpretation of findings within studies as well as the synthesis of data across studies. Moreover, recall periods warrant consideration regarding the likelihood of accurately recalling presenteeism over longer periods (e.g. 12 months) and the impact of seasonal events and holiday periods on ‘typical’ ATOD use, especially if they fall within shorter recall periods of days and weeks. For instance, whilst there is no single suggested recall period for all phenomena [98], one study advocates for the use of a two-week recall period for presenteeism to avoid recall bias, assuming that the previous two weeks would be an accurate reflection of presenteeism over a typical year and extrapolated accordingly [99]. Similarly, short recall periods of one week have been proposed for alcohol consumption [100], although 12-month periods may accommodate seasonal variation and intermittent or episodic drinking [101]. However, the short-, medium-, and long-term health implications of different substances need to be taken into account, hence questions about tobacco often using lifetime recall periods, although the recall of tobacco use is largely considered reliable [102]. In a systematic review of the association of alcohol with presenteeism, Thørrisen et al. [7] similarly found heterogeneity across studies that complicated meta-analyses. Overall, findings from this scoping review suggest that there is substantial scope for the use of validated instruments and consistent approaches to be standardised for determining presenteeism related to all types of substances used by workers that may impact presenteeism and productivity.

## Strengths, limitations, and future research

The inclusion of the LILACS database increased the search scope beyond English language databases. However, other ATOD-related presenteeism instruments may exist in other formats and languages that were unidentifiable through the search protocol outlined here, especially unpublished instruments used by organisations. The aims of this scoping review were not concerned with different types of illicit substances. However, increasing legalisation and acceptability of ‘de-stigmatising products’ [103] like recreational and medicinal cannabis suggest that a review of approaches to determining the impact of cannabis on productivity may be worthwhile.

More broadly, further research is also required to determine how the different approaches to measuring and reporting ATOD-related presenteeism identified in this scoping review impact the production and interpretation of data. This could involve a systematic review of measurement properties of the validated instruments identified in this review by applying COSMIN (COnsensus-based Standards for the selection of health status Measurement Instruments) [104]. Such determinations will be important for providing a basis for future recommendations about approach (direct versus indirect), instrument selection, recall periods, and what considerations apply to different types of substances and their legal status (licit/illicit). At the same time, a similar scoping review of ATOD-related absenteeism is needed to determine if there is a similar preference for indirect approaches and diversity across instruments, recall periods, and reporting.

Not all studies provided information describing type of worker or industry, which made it difficult to identify differences between safety and non-safety-sensitive industries. In addition, one study did not describe the instrument to determine presenteeism [45], and five did not describe the instruments used to determine ATOD use [29, 35–37, 51]. Moreover, studies did not provide sufficient detail about workplace culture that could enable the identification of trends for risk-averse or health-based [105], recovery-friendly [106] workplaces. This information provides important context, as organisational culture can support or undermine workers’ recovery from substance use disorders and impact addiction disclosure [107]. The consistent inclusion of this information in studies reporting ATOD-related presenteeism is recommended to improve the transparency of reporting practices and enhance the value of future reviews.

## Conclusion

This scoping review was the first to consider which instruments and approaches have been used across original studies to determine ATOD-related presenteeism. An indirect approach was most common, which involved comparing differences in work performance between people who used ATOD and those who did not. Direct approaches asking workers to report on presenteeism directly attributable to their ATOD use may have been less common as a deliberate attempt to mitigate stigma, social desirability bias, and workers’ concerns for legal or occupational repercussions. There was little consistency across the instruments used for both approaches. Where detail was provided, a variety of validated and non-validated instruments, recall periods, and ways of reporting findings was observed across the studies. This variability may undermine the validity of data and complicate the interpretation of findings within studies using an indirect approach as well as comparisons across all types of studies. Further research is needed to determine the impact on data quality of the different instruments and approaches identified in this scoping review, which could inform recommendations for a standardised approach to determining ATOD-related presenteeism. In turn, this will support a more robust understanding of the impact of ATOD-related presenteeism in relation to different industries, occupations, and substances which is necessary to design and evaluate tailored workplace ATOD interventions that support worker health, safety, and productivity.

## Data Availability

No datasets were generated or analysed during the current study.

## References

[1] World Health Organization. Global status report on alcohol and health and treatment of substance use disorders. Geneva: Switzerland; 2024.

[2] Rehm J, Baliunas D, Borges GL, Graham K, Irving H, Kehoe T, et al. The relation between different dimensions of alcohol consumption and burden of disease: an overview. Addiction. 2010;105(5):817–43. 20331573 10.1111/j.1360-0443.2010.02899.x PMC3306013

[3] He H, Pan Z, Wu J, Hu C, Bai L, Lyu J. Health effects of tobacco at the global, regional, and national levels: results from the 2019 global burden of disease study. Nicotine Tob Res. 2022;24(6):864–70. 34928373 10.1093/ntr/ntab265

[4] Larsson SC, Burgess S. Appraising the causal role of smoking in multiple diseases: a systematic review and meta-analysis of Mendelian randomization studies. EBioMedicine. 2022;82:104154. 35816897 10.1016/j.ebiom.2022.104154 PMC9278068

[5] Roche AM, Chapman J, Duraisingam V, Phillips B, Finnane J, Pidd K. Construction workers’ alcohol use, knowledge, perceptions of risk and workplace norms. Drug Alcohol Rev. 2020;39(7):941–9. 32350917 10.1111/dar.13075

[6] Medina-Martinez J, Alino M, Vazquez-Martinez A, Villanueva-Blasco VJ, Cano-Lopez I. Risk and protective factors associated with drug use in healthcare professionals: a systematic review. J Psychoactive Drugs. 2024;56(3):397–411. 37341709 10.1080/02791072.2023.2227173

[7] Thørrisen MM, Bonsaksen T, Hashemi N, Kjeken I, van Mechelen W, Aas RW. Association between alcohol consumption and impaired work performance (presenteeism): a systematic review. BMJ Open. 2019;9(7):e029184. 31315869 10.1136/bmjopen-2019-029184 PMC6661906

[8] Pidd K, Berry J, Harrison J, Roche A, Driscoll T, Newson R. Alcohol and work: patterns of use, workplace culture and safety. (Injury Research and Statistics Series; no.: 28). Canberra: Australian Institute of Health and Welfare; 2006. https://www.aihw.gov.au/reports/alcohol/alcohol-use-workplace-culture-safety/contents/executive-summary. Accessed 11 November 2024.

[9] Halpern MT, Shikiar R, Rentz AM, Khan ZM. Impact of smoking status on workplace absenteeism and productivity. Tob Control. 2001;10(3):233–8. 11544387 10.1136/tc.10.3.233 PMC1747570

[10] Parrott S, Godfrey C, Raw M. Costs of employee smoking in the workplace in Scotland. Tob Control. 2000;9(2):187–92. 10841855 10.1136/tc.9.2.187 PMC1748323

[11] Dinis-Oliveira RJ, Magalhães T. Abuse of Licit and Illicit psychoactive substances in the workplace: medical, toxicological, and forensic aspects. J Clin Med. 2020;9(3):770. 32178358 10.3390/jcm9030770 PMC7141377

[12] Whetton S, Tait R, Gilmore W, Dey T, Agramunt S, Halim SA, et al. Examining the social and economic costs of alcohol use in Australia: 2017/18. Perth, WA: National Drug Research Institute, Curtin University; 2021. Report No.: 064873675X.

[13] Severeijns NR, Sips ASM, Merlo A, Bruce G, Verster JC. Absenteeism, presenteeism, and the economic costs of alcohol hangover in the netherlands. Healthcare (Basel, Switzerland). 2024;12(3):335. 38338220 10.3390/healthcare12030335 PMC10855845

[14] Shockey TM, Esser MB. Binge drinking by occupation groups among currently employed U.S. adults in 32 states, 2013–2016. Subst Use Misuse. 2020;55(12):1968–79. 32619144 10.1080/10826084.2020.1784947 PMC8725193

[15] Buvik K, Moan IS, Halkjelsvik T. Alcohol-related absence and presenteeism: beyond productivity loss. Int J Drug Policy. 2018;58:71–7. 29864644 10.1016/j.drugpo.2018.05.005

[16] Jonhes KS, Casswell S, Zhang J-F. The economic costs of alcohol-related absenteeism and reduced productivity among the working population of New Zealand. Addiction. 1995;90(11):1455–61. 8528030 10.1046/j.1360-0443.1995.901114553.x

[17] Di Censo G, Thompson K, Bowden J. Alcohol and other drug use by Australian workers: insights from a nationally representative cross-sectional survey. Health Promot Int. 2025;40(2):048. 10.1093/heapro/daaf048 PMC12008742 40252001

[18] McEntee A, Pointer, S., Pincombe, A., Nicholas, R. and Bowden, J. . Alcohol and other drug use: A focus on employed Australians: Part 1: Prevalence and consequences. Adelaide, South Australia: National Centre for Education and Training on Addiction (NCETA), Flinders Health and Medical Research Institute (FHMRI), Flinders University; 2023.

[19] Owen AJ, Maulida SB, Zomer E, Liew D. Productivity burden of smoking in Australia: a life table modelling study. Tob Control. 2019;28(3):297–304. 30012640 10.1136/tobaccocontrol-2018-054263 PMC6580760

[20] Dew K, Keefe V, Small K. ‘Choosing’ to work when sick: workplace presenteeism. Soc Sci Med. 2005;60(10):2273–82. 15748675 10.1016/j.socscimed.2004.10.022

[21] Garrow V. Presenteeism: A Review of Current Thinking2016 27 March 2025. http://www.employment-studies.co.uk/system/files/resources/files/507_0.pdf.

[22] Biron C, Brun JP, Ivers H, Cooper C. At work but ill: psychosocial work environment and well-being determinants of presenteeism propensity. J Public Ment Health. 2006;5(4):26–37.

[23] Sullivan T, Edgar F, McAndrew I. The hidden costs of employee drinking: a quantitative analysis. Drug Alcohol Rev. 2019;38(5):543–53. 31170328 10.1111/dar.12935

[24] Kinman G. Sickness presenteeism at work: prevalence, costs and management. Br Med Bull. 2019;129(1):69–78. 30649219 10.1093/bmb/ldy043

[25] Ospina MB, Dennett L, Waye A, Jacobs P, Thompson AH. A systematic review of measurement properties of instruments assessing presenteeism. Am J Manag Care. 2015;21(2):e171–85. 25880491

[26] Maestas NA, Mullen KJ, Rennane S. Absenteeism and presenteeism among American workers. J Disabil Policy Stud. 2021;32(1):13–23.

[27] McGregor A, Caputi P. Measuring Presenteeism. In: McGregor A, Caputi P, editors. Presenteeism behaviour: current research, theory and future directions. Cham: Springer International Publishing; 2022. p. 25–50.

[28] Aas RW, Haveraaen L, Sagvaag H, Thørrisen MM. The influence of alcohol consumption on sickness presenteeism and impaired daily activities. The WIRUS Screening Study. PLoS ONE. 2017;12(10):e0186503. 29040323 10.1371/journal.pone.0186503 PMC5645115

[29] Baker CL, Flores NM, Zou KH, Bruno M, Harrison VJ. Benefits of quitting smoking on work productivity and activity impairment in the United States, the European Union and China. Int J Clin Pract. 2017;71(1):e12900. 28097760 10.1111/ijcp.12900 PMC5299499

[30] Baker CL, Bruno M, Emir B, Li VW, Goren A. Smoking cessation is associated with lower indirect costs. J Occup Environ Med. 2018;60(6):490–5. 29465514 10.1097/JOM.0000000000001302 PMC5991186

[31] Bonsaksen T, Thørrisen MM, Skogen JC, Hesse M, Aas RW. Are demanding job situations associated with alcohol-related presenteeism? The WIRUS-screening study. Int J Environ Res Public Health. 2021;18(11):6169. 34200397 10.3390/ijerph18116169 PMC8201186

[32] Bunn Iii WB, Stave GM, Downs KE, Alvir JMJ, Dirani R. Effect of smoking status on productivity loss. J Occup Environ Med. 2006;48(10):1099–108. 17033509 10.1097/01.jom.0000243406.08419.74

[33] França MH, Pereira FG, Wang YP, Andrade LH, Alonso J, Viana MC. Individual and population level estimates of work loss and related economic costs due to mental and substance use disorders in Metropolitan São Paulo. Brazil J Affect Disord. 2022;296:198–207. 34610514 10.1016/j.jad.2021.09.070

[34] Goetzel RZ, Carls GS, Wang S, Kelly E, Mauceri E, Columbus D, et al. The relationship between modifiable health risk factors and medical expenditures, absenteeism, short-term disability, and presenteeism among employees at novartis. J Occup Environ Med. 2009;51(4):487–99. 19337132 10.1097/JOM.0b013e31819eb902

[35] Haglund E, Petersson IF, Bremander A, Bergman S. Predictors of presenteeism and activity impairment outside work in patients with spondyloarthritis. J Occup Rehabil. 2015;25(2):288–95. 25173795 10.1007/s10926-014-9537-2

[36] Henke RM, Ellsworth D, Wier L, Snowdon J. Opioid use disorder and employee work presenteeism, absences, and health care costs. J Occup Environ Med. 2020;62(5):344–9. 32049873 10.1097/JOM.0000000000001830

[37] Holden L, Scuffham PA, Hilton MF, Ware RS, Vecchio N, Whiteford HA. Which health conditions impact on productivity in working Australians? J Occup Environ Med. 2011;53(3):253–7. 21346633 10.1097/JOM.0b013e31820d1007

[38] Lee SY, Lee J, Kwon M. Impacts of heavy smoking and alcohol consumption on workplace presenteeism: a cross-sectional study. Medicine (Baltimore). 2021;100(47):e27751. 34964731 10.1097/MD.0000000000027751 PMC8615302

[39] Musich S, Hook D, Baaner S, Spooner M, Edington DW. The association of corporate work environment factors, health risks, and medical conditions with Presenteeism among Australian employees. Am J Health Promot. 2006;21(2):127–36. 17152252 10.4278/0890-1171-21.2.127

[40] Otsubo T, Kinjo A, Kuwabara Y, Hongja K, Osaki Y. Lifestyle factors associated with presenteeism among city government office workers: a cross-sectional study. J Occup Health. 2024;66(1):uiad012. 38258943 10.1093/JOCCUH/uiad012 PMC11465367

[41] Pelletier B, Boles M, Lynch W. Change in health risks and work productivity over time. J Occup Environ Med. 2004;46(7):746–54. 15247815 10.1097/01.jom.0000131920.74668.e1

[42] Santos LAd. Atividade física e morbidade cardiovascular referidas pelos gerentes e diretores de uma indústria automobilística: influência de um programa de condicionamento físico supervisionado. (Physical activity and cardiovascular morbidity reported by managers and directors of an automotive industry: The influence of a supervised physical conditioning program). Unpublished PhD Dissertation. São Paulo, Brasil: Universidade de São Paulo; 2008.

[43] Sherman BW, Lynch WD. The relationship between smoking and health care, workers’ compensation, and productivity costs for a large employer. J Occup Environ Med. 2013;55(8):879–84. 23924829 10.1097/JOM.0b013e31829f3129

[44] Shiratsuchi D, Motohiro A, Okuyama K, Abe T. Relationship between occupational stress and presenteeism status among workers in small and medium-sized enterprises. Arch Environ Occup Health. 2024;79(2):83–90. 38829113 10.1080/19338244.2024.2359409

[45] Shrestha SS, Ghimire R, Wang X, Trivers KF, Homa DM, Armour BS. Cost of cigarette smoking-attributable productivity losses, U.S., 2018. Am J Prev Med. 2022;63(4):478–85. 35909028 10.1016/j.amepre.2022.04.032 PMC10108669

[46] Suwa K, Flores NM, Yoshikawa R, Goto R, Vietri J, Igarashi A. Examining the association of smoking with work productivity and associated costs in Japan. J Med Econ. 2017;20(9):938–44. 28685629 10.1080/13696998.2017.1352507

[47] Thompson AH. Measures of mental health and addictions conditions show a U-shaped relationship with self-rated worker performance. Soc Psychiatry Psychiatr Epidemiol. 2021;56(10):1823–33. 32542463 10.1007/s00127-020-01894-w

[48] Tran BX, Nguyen LH, Nguyen CT, Latkin CA. Health-related work productivity loss is low for patients in a methadone maintenance program in Vietnam. Int J Drug Policy. 2018;60:1–7. 30077903 10.1016/j.drugpo.2018.07.007

[49] Tsuchida M, Monma T, Ozawa S, Kikuchi A, Takeda F. Lifestyle habit change related to presenteeism change among Japanese employees. AIMS Public Health. 2024;11(3):729–46. 39416900 10.3934/publichealth.2024037 PMC11474334

[50] Williden M, Schofield G, Duncan S. Establishing links between health and productivity in the New Zealand workforce. J Occup Environ Med. 2012;54(5):545–50. 22547124 10.1097/JOM.0b013e31824fe0c8

[51] Won Y, Kim HC, Kim J, Kim M, Yang SC, Park SG, et al. Impacts of presenteeism on work-related injury absence and disease absence. Annals Occup Environ Med. 2022;34(1):e25. 10.35371/aoem.2022.34.e25 PMC9560897 36267359

[52] Peters MDJ, Marnie C, Tricco AC, Pollock D, Munn Z, Alexander L, et al. Updated methodological guidance for the conduct of scoping reviews. JBI Evid Synth. 2020;18(10):2119–26. 33038124 10.11124/JBIES-20-00167

[53] Peters MD, Godfrey CM, McInerney P, Soares CB, Khalil H, Parker D. The Joanna Briggs Institute reviewers’ manual 2015: methodology for JBI scoping reviews. 2015.

[54] Popay J, Roberts H, Sowden A, Petticrew M, Arai L, Rodgers M, et al. Guidance on the conduct of narrative synthesis in systematic reviews. A Product from the ESRC Methods Programme Version. 2006;1(1):b92.

[55] The World Bank. World Bank country classifications by income level for 2024–20252025. https://datacatalogapi.worldbank.org/ddhxext/ResourceDownload?resource_unique_id=DR0090755.

[56] de Sousa RM, Ribeiro AC, Valim MD. Burnout syndrome, presenteeism and loss of productivity in nursing workers. Revista de Enfermagem Referencia. 2023;6(2):1–10.

[57] Reilly MC, Zbrozek AS, Dukes EM. The validity and reproducibility of a work productivity and activity impairment instrument. Pharmacoeconomics. 1993;4(5):353–65. 10146874 10.2165/00019053-199304050-00006

[58] Roijen H-vR, Essink-Bot M-L. The health and labour questionnaire manual. Erasmus University, Rotterdam; 2000. http://hdl.handle.net/1765/1313.

[59] Bhattacharya A. Financial headache: the cost of workplace hangovers and intoxication to the UK economy; 2019. https://www.ias.org.uk/wp-content/uploads/2020/06/rp35062019.pdf. Accessed 14 January 2025.

[60] Kessler RC, Barber C, Beck A, Berglund P, Cleary PD, McKenas D, et al. The World Health Organization Health and work performance Questionnaire (HPQ). J Occup Environ Med. 2003;45(2):156–74. 12625231 10.1097/01.jom.0000052967.43131.51

[61] Kessler RC, Ustün TB. The World Mental Health (WMH) Survey Initiative Version of the World Health Organization (WHO) Composite International Diagnostic Interview (CIDI). Int J Methods Psychiatr Res. 2004;13(2):93–121. 15297906 10.1002/mpr.168 PMC6878592

[62] Kawakami N, Inoue A, Tsuchiya M, Watanabe K, Imamura K, Iida M, et al. Construct validity and test-retest reliability of the World Mental Health Japan version of the World Health Organization Health and Work Performance Questionnaire Short Version: a preliminary study. Ind Health. 2020;58(4):375–87. 32173661 10.2486/indhealth.2019-0090 PMC7417506

[63] Ciconelli RM, Soárez PCD, Kowalski CCG, Ferraz MB. The Brazilian Portuguese version of the work productivity and activity impairment: general health (WPAI-GH) questionnaire. São Paulo Med J. 2006;124:325–32. 17322953 10.1590/S1516-31802006000600005 PMC11068280

[64] Lerner D, Amick BCI, Rogers WH, Malspeis S, Bungay K, Cynn D. The work limitations questionnaire. Med Care. 2001;39(1):72–85. 11176545 10.1097/00005650-200101000-00009

[65] Walker TJ, Tullar JM, Diamond PM, Kohl HW 3rd, Amick BC 3rd. Validity and reliability of the 8-item work limitations questionnaire. J Occup Rehabil. 2017;27(4):576–83. 28025750 10.1007/s10926-016-9687-5 PMC5484749

[66] Cho Y. Data resource profile: the Korean Working Conditions Survey (KWCS). Ann Occup Environ Med. 2023;35:e49. 38148917 10.35371/aoem.2023.35.e49 PMC10751213

[67] Kim YS, Rhee KY, Oh MJ, Park J. The validity and reliability of the second Korean working conditions survey. Saf Health Work. 2013;4(2):111–6. 23961335 10.1016/j.shaw.2013.05.001 PMC3732146

[68] Subramaniam M, Abdin E, Vaingankar JA, Sagayadevan V, Shahwan S, Picco L, et al. Validation of the World Health Organization disability assessment schedule 2.0 among older adults in an Asian country. Singapore Med J. 2020;61(5):246–53. 31197373 10.11622/smedj.2019049 PMC7905154

[69] Goetzel RZ, Ozminkowski RJ, Long SR. Development and reliability analysis of the Work Productivity Short Inventory (WPSI) instrument measuring employee health and productivity. J Occup Environ Med. 2003;45(7):743–62. 12855915 10.1097/01.jom.0000079085.95532.32

[70] Ozminkowski RJ, Goetzel RZ, Long SR. A validity analysis of the Work Productivity Short Inventory (WPSI) instrument measuring employee health and productivity. J Occup Environ Med. 2003;45(11):1183–95. 14610400 10.1097/01.jom.0000091694.62216.64

[71] Robbins L, Hall J. How to Practice Prospective Medicine. Indianapolis, IN: Methodist Hospital of Indiana; 1970.

[72] Edington DW, Yen L, Braunstein A. The reliability and validity of HRAs. In: Hyner GC, Peterson KW, Travis JW, Dewey JE, Foerster JJ, Framer EM, editors. SPM Handbook of Health Assessment Tools Fourth ed. Pittsburgh, PA: The Society of Prospective Medicine and The Institute for Health and Productivity Management; 1999. p. 135–42.

[73] Saunders JB, Aasland OG, Babor TF, De la Fuente JR, Grant M. Development of the alcohol use disorders identification test (AUDIT): WHO collaborative project on early detection of persons with harmful alcohol consumption-II. Addiction. 1993;88(6):791–804. 8329970 10.1111/j.1360-0443.1993.tb02093.x

[74] Bush K, Kivlahan DR, McDonell MB, Fihn SD, Bradley KA, Project ACQI. The AUDIT alcohol consumption questions (AUDIT-C): An effective brief screening test for problem drinking. Arch Internal Med. 1998;158(16):1789–95. 9738608 10.1001/archinte.158.16.1789

[75] van Gils Y, Franck E, Dierckx E, van Alphen SPJ, Saunders JB, Dom G. Validation of the AUDIT and AUDIT-C for hazardous drinking in community-dwelling older adults. Int J Environ Res Public Health. 2021;18(17):9266. 34501856 10.3390/ijerph18179266 PMC8431181

[76] Stewart RE, Cardamone NC, Schachter A, Becker C, McKay JR, Becker-Haimes EM. A systematic review of brief, freely accessible, and valid self-report measures for substance use disorders and treatment. Drug Alcohol Depend. 2023;243:109729. 36535096 10.1016/j.drugalcdep.2022.109729 PMC9872256

[77] Ewing JA. Detecting alcoholism: the CAGE questionnaire. JAMA. 1984;252(14):1905–7. 6471323 10.1001/jama.252.14.1905

[78] Skinner HA. The drug abuse screening test. Addict Behav. 1982;7(4):363–71. 7183189 10.1016/0306-4603(82)90005-3

[79] Yudko E, Lozhkina O, Fouts A. A comprehensive review of the psychometric properties of the Drug Abuse Screening Test. J Subst Abuse Treat. 2007;32(2):189–98. 17306727 10.1016/j.jsat.2006.08.002

[80] Thompson AH, Jacobs P, Dewa C. The Alberta Survey of addictive behaviours and mental health in the workforce: 2009. 2011. https://www.ihe.ca/advanced-search/the-alberta-survey-of-addictive-behaviours-and-mental-health-in-the-workforce-2009. Accessed 11 April 2025.

[81] Swarbrick M, Di Bello A, Eissenstat SJ, Nemec PB, Hien DA, Gill KJ. Factor structure, reliability, and construct validity of the wellness inventory. Psychiatr Serv. 2024;76(3):263–9. 10.1176/appi.ps.20230622 39497530

[82] Centers for Disease Control and Prevention. Behavioral Risk Factor Surveillance System. n.d.

[83] Pierannunzi C, Hu SS, Balluz L. A systematic review of publications assessing reliability and validity of the Behavioral Risk Factor Surveillance System (BRFSS), 2004–2011. BMC Med Res Methodol. 2013;13:1–14. 23522349 10.1186/1471-2288-13-49 PMC3622569

[84] Gelaye B, Williams MA, Lemma S, Deyessa N, Bahretibeb Y, Shibre T, et al. Diagnostic validity of the composite international diagnostic interview (CIDI) depression module in an East African population. Int J Psychiatry Med. 2013;46(4):387–405. 24922989 10.2190/PM.46.4.e PMC4058648

[85] National Centre for Health Statistics. Plan and operation of the third National Health and Nutrition Examination Survey, 1988–1994: Reference Manuals and Reports: Weighting and Estimation Methodology Report. Hyattsville, MD: US Dept of Health & Human Services, Public Health Service, Centers for Disease Control & Prevention; 1994.

[86] Gerritsen S, Stefanogiannis N, Galloway Y, Devlin M, Templeton R, Yeh L. A portrait of health: Key results of the 2006/07 New Zealand health survey. Wellington: Ministry of Health; 2008.

[87] CfDC, Prevention. National Health Interview Survey, 1997–2018. Survey description document (Accessed 15 Oct 2021, at https://www.cdcgov/nchs/nhis/1997-2018.htm). 2021.

[88] Ministry of Health Labor and Welfare of Japan. Standard program of the specific health checkup and guidance. 2013.

[89] Kantar Health. US National Health and Wellness Survey (NHWS). New York, USA: Kantar Health, ; n.d.

[90] McNeil SR. Understanding substance use stigma. J Soc Work Pract Addict. 2021;21(1):83–96.

[91] Roche A, Kostadinov V, Pidd K. The stigma of addiction in the workplace. In: Avery JD, Avery JJ, editors. The stigma of addiction: An essential guide. Cham, Switzerland: Springer; 2019. p. 167–99.

[92] Schulte B, O’Donnell AJ, Kastner S, Schmidt CS, Schäfer I, Reimer J. Alcohol screening and brief intervention in workplace settings and social services: a comparison of literature. Front Psych. 2014;5:131. 10.3389/fpsyt.2014.00131 PMC4186263 25339914

[93] Watson H, Godfrey C, McFadyen A, McArthur K, Stevenson M, Holloway A. Screening and brief intervention delivery in the workplace to reduce alcohol-related harm: a pilot randomized controlled trial. Int J Nurs Stud. 2015;52(1):39–48. 25062806 10.1016/j.ijnurstu.2014.06.013

[94] Cook RF, Hersch RK, McPherson TL. Drug assessment methods in the workplace. Drug testing technology. Boca Raton: CRC Press; 2020. p. 255–81.

[95] World Health Organisation ASSIST Working Group. The alcohol, smoking and substance involvement screening test (ASSIST): development, reliability and feasibility. Addiction. 2002;97(9):1183–94. 12199834 10.1046/j.1360-0443.2002.00185.x

[96] Ali R, Meena S, Eastwood B, Richards I, Marsden J. Ultra-rapid screening for substance-use disorders: the Alcohol, Smoking and Substance Involvement Screening Test (ASSIST-Lite). Drug Alcohol Depend. 2013;132(1–2):352–61. 23561823 10.1016/j.drugalcdep.2013.03.001

[97] Rmadi N, Kotti N, Masmoudi R, Dhouib F, Hammami KJ, Masmoudi ML, et al. Impact of addictive behaviors on productivity at work among employees working on an onshore oil field. Eur Psychiat. 2021;64(S1):S564-S.

[98] Stull DE, Leidy NK, Parasuraman B, Chassany O. Optimal recall periods for patient-reported outcomes: challenges and potential solutions. Curr Med Res Opin. 2009;25(4):929–42. 19257798 10.1185/03007990902774765

[99] Allen D, Hines EW, Pazdernik V, Konecny LT, Breitenbach E. Four-year review of presenteeism data among employees of a large United States health care system: a retrospective prevalence study. Hum Resour Health. 2018;16(1):59. 30413168 10.1186/s12960-018-0321-9 PMC6234777

[100] Ekholm O, Strandberg-Larsen K, Grønbæk M. Influence of the recall period on a beverage-specific weekly drinking measure for alcohol intake. Eur J Clin Nutr. 2011;65(4):520–5. 21326272 10.1038/ejcn.2011.1

[101] Tevik K, Bergh S, Selbæk G, Johannessen A, Helvik A-S. A systematic review of self-report measures used in epidemiological studies to assess alcohol consumption among older adults. PLoS ONE. 2021;16(12):e0261292. 34914759 10.1371/journal.pone.0261292 PMC8675766

[102] Brigham J, Lessov-Schlaggar CN, Javitz HS, McElroy M, Krasnow R, Swan GE. Reliability of adult retrospective recall of lifetime tobacco use. Nicotine Tob Res. 2008;10(2):287–99. 18236293 10.1080/14622200701825718

[103] Simkins TJ, Allen B. Illicit to legal: marijuana as a de-stigmatising product and the role of social acceptability in new product adoption. Innovation. 2020;22(4):447–68.

[104] Mokkink LB, Terwee CB, Knol DL, Stratford PW, Alonso J, Patrick DL, et al. Protocol of the COSMIN study: consensus-based Standards for the selection of health measurement instruments. BMC Med Res Methodol. 2006;6:1–7. 16433905 10.1186/1471-2288-6-2 PMC1368990

[105] Goetzel RZ, Henke RM, Tabrizi M, Pelletier KR, Loeppke R, Ballard DW, et al. Do workplace health promotion (wellness) programs work? J Occup Environ Med. 2014;56(9):927–34. 25153303 10.1097/JOM.0000000000000276

[106] Frone MR, Chosewood LC, Osborne JC, Howard JJ. Workplace supported recovery from substance use disorders: defining the construct, developing a model, and proposing an agenda for future research. Occup Health Sci. 2022;6(4):475–511. 37206918 10.1007/s41542-022-00123-x PMC10193449

[107] Burns VF, Walsh CA, Smith J. A qualitative exploration of addiction disclosure and stigma among faculty members in a Canadian university context. Int J Environ Res Public Health. 2021;18(14):7274. 34299723 10.3390/ijerph18147274 PMC8306368

