# Correction: Present but Overlooked: A Scoping Review of Instruments and Approaches for Measuring Presenteeism Related to Alcohol, Tobacco, and Drug Use

**DOI:** 10.1007/s10926-025-10324-0

**Published:** 2025-09-01

**Authors:** Kirrilly Thompson, Md Abdul Ahad, Gianluca Di Censo, Sonia Hines, Nicholas Rich, Alice McEntee, Jacqueline Bowden

**Affiliations:** 1 The National Centre for Education and Training on Addiction, Flinders University, Adelaide, South Australia Australia; 2 College of Medicine and Public Health, Flinders Health and Medical Research Institute, Flinders University, Adelaide, SA 5000 Australia; 3 School of Medicine and Public Health, College of Health, Medicine and Wellbeing, University of Newcastle, Callaghan, NSW 2308 Australia; 4 Department of Rural Sociology and Development, Sylhet Agricultural University, Sylhet, 3100 Bangladesh; 5 The University of Adelaide, JBI, School of Public Health, Adelaide, South Australia Australia

**Correction to: Journal of Occupational Rehabilitation (2025)** 10.1007/s10926-025-10317-z

In this article, in the subheadings “Study type, design, and sample size” and “Instruments used to determine ATOD use” incorrect reference citations have been corrected due to a production error, and this has been explained in the below points.

1. In the subheading “Study type, design, and sample size”, in the sentence stating “Almost all (*n* = 26, 96%) studies……in English [13, 15, 23, 28–41, 43–51, 56]”, the reference citation number “56” was incorrectly listed and should have been deleted.
2. In the subheading “Study type, design, and sample size”, in the sentence starting “Over one quarter of studies….. national survey data sources [29, 30, 38, 40, 43, 45, 46, 49]” the reference citation number “40” was incorrectly listed and should have been replaced with “36”, and “43” should not have been included.
3. In the subheading “Instruments used to determine ATOD use” in second paragraph, in the sentence ending “…past 12 months [48]” the reference citation number was incorrectly listed and should have been replaced with “[47]”.

The original article has been corrected.

